# Association between increased arterial stiffness and clinical outcomes in patients with early sepsis: a prospective observational cohort study

**DOI:** 10.1186/s40635-019-0252-3

**Published:** 2019-05-16

**Authors:** Sigita Kazune, Andris Grabovskis, Corrado Cescon, Eva Strike, Indulis Vanags

**Affiliations:** 1 Department of Anesthesiology, Hospital of Traumatology and Orthopedics, 22 Duntes Street, Riga, LV-1013 Latvia; 2 Institute of Atomic Physics and Spectroscopy, University of Latvia, Riga, Latvia; 3 Department of Anesthesiology and Cardiac Surgery, Pauls Stradins Clinical University Hospital, Riga, Latvia; 4 Department of Anesthesiology and Reanimatology, Riga Stradins University, Riga, Latvia; 5 Vacallo, Switzerland

**Keywords:** Sepsis, Hemodynamics, Pulse wave velocity, Prognostic factor

## Abstract

**Background:**

Conduit arteries, especially the aorta, play a major role in ensuring efficient cardiac function and optimal microvascular flow due to their viscoelastic properties. Studies in animals and on isolated arteries show that acute systemic inflammation can cause aortic stiffening which affects hemodynamic efficiency. Carotid-femoral pulse wave velocity, a measure of aortic stiffness, may be useful as a bedside investigational method in patients with early sepsis admitted to intensive care, as circulatory changes can lead to multiple organ failure and increased mortality. This study aims to investigate arterial stiffness in early sepsis and its association with clinical outcomes.

**Methods:**

This prospective observational study included adult patients with severe sepsis or septic shock admitted to our intensive care unit (*n* = 45). Their carotid-femoral pulse wave velocity was measured within 24 h of admission. We assessed the progression of multiple organ as well as cardiovascular failure by sequential SOFA scores. Prediction models for the progression of multiple organ and cardiovascular failure were constructed using multivariate logistic regression with pulse wave velocity and vasopressor use as predictors. A Cox proportional hazards model was used to examine the relationship between pulse wave velocity and survival time.

**Results:**

The median pulse wave velocity for the cohort was 14.6 (8.1–24.7) m/s. There was no association between pulse wave velocity and the progression of multiple organ failure, before or after adjustment for vasopressor use. No association was found between pulse wave velocity and subsequent improvement in cardiovascular failure in the subgroup of patients who had cardiovascular instability at baseline. Cox regression and survival analyses with age, APACHE II, and baseline SOFA as confounders showed a shorter hospital survival time for patients with pulse wave velocity > 24.7 m/s (HR = 9.45, 95% CI 1.24–72.2; *P* = 0.03).

**Conclusions:**

Patients with severe sepsis and septic shock admitted to intensive care have higher arterial stiffness than in the general population. No convincing association was found between pulse wave velocity at admission and the progression of multiple organ or cardiovascular failure, although the group with pulse wave velocity > 24.7 m/s had shorter survival time.

**Electronic supplementary material:**

The online version of this article (10.1186/s40635-019-0252-3) contains supplementary material, which is available to authorized users.

## Background

Sepsis, a syndrome defined as the host’s dysfunctional response to infection, is a frequent reason for admission to intensive care unit [1]. A major component of sepsis pathophysiology is a vascular injury with an imbalance of vasodilatory and vasoconstrictive mechanisms, which might lead to abnormal perfusion of the vital organs and multiple organ failure [2]. Traditionally, research into mechanisms underlying macro-hemodynamic alterations has focused on sepsis-induced myocardial depression and vasodilation of peripheral vascular beds [3]. Yet, the mechanical properties of conduit arteries, especially the aorta, also play a major role in accommodating the blood ejected during systole and in ensuring both efficient cardiac function and optimal flow conditions in the peripheral vasculature [4].

Most of the current knowledge about the effects of acute systemic inflammation on the aorta comes from experimental research on animals or isolated arteries [5–7]. Even a single injection of endotoxin in laboratory animals causes endothelial denudation in the aorta and impaired endothelium-dependent vasodilation [5]. There have been efforts to reproduce results from in vitro research to human studies using carotid-femoral pulse wave velocity (PWV) as a measure of viscoelastic properties of arteries. Carotid-femoral PWV is the speed of travel of the pressure wave along the aorta. Its measurement is non-invasive, can be done by the bedside without a proprietary device, is based on published reference values, and has multiple studies supporting its use in predicting mortality in various chronic conditions [8–10]. Most of the populations studied were either healthy individuals with low-grade inflammation [11] or patients with chronic inflammatory disease, such as rheumatoid arthritis [12]. An increase in PWV has also been found to be associated with markers of acute inflammation, such as white cell count and levels of C-reactive protein [13, 14].

In a previous study, we investigated the carotid-femoral PWV as a tool for the assessment of conduit artery stiffness during the resuscitation phase of sepsis, but the results did not support the role of PWV as a prognostic factor [15]. However, the patient cohort was small and included patients with arrhythmias who can show biased measurements because of uneven RR intervals. The side of measurement was not defined, and a mixture of right- and left-sided measurements was reported which limits the validity of the study. The aims of this study were to reassess carotid-femoral PWV in a larger cohort of patients with sepsis within 24 h of admission to the intensive care unit and to examine the association between PWV at admission and the progression of multiple organ and cardiovascular failure.

## Materials and methods

### Study design and population

After approval by the Institutional Ethics Committee of Riga Stradins University, we conducted a prospective observational cohort study. Written informed consent from the patient or their next of kin was obtained before being included in the study.

Between September 2015 and October 2016, 164 consecutive adult (> 18 years of age) patients with severe sepsis or septic shock who were admitted to our mixed 16-bed intensive care unit (ICU) within the last 24 h were found eligible for inclusion. Readmitted patients were eligible for inclusion only during their first episode of sepsis. Severe sepsis and septic shock were defined according to the American College of Chest Physicians/Society of Critical Care Medicine Consensus Conference (1992) criteria [16]. Exclusion criteria were pregnancy, arrhythmias, previous aortic surgery, and administration of nitrates. The study involved the recording of pulse waves using originally designed photoplethysmograph; therefore, patients could be recruited only when an investigator was available.

The demographic and clinical variables included age, sex, length of hospital stay, the primary site and type of infection, and physiologic and treatment variables necessary for the calculation of Acute Physiology and Chronic Health Evaluation (APACHE) II [17] and baseline Sequential Organ Failure Assessment (SOFA) scores [18]. In mechanically ventilated patients, the last known value of the verbal component of the Glasgow Coma Score before tracheal intubation was used until the patient was able to speak after extubation. Information regarding their history of smoking, previous cardiovascular disease, and diabetes mellitus was obtained from the patients or their medical records.

Clinicians not involved in the study treated the patients according to local protocols outlining the use of antibiotics, vasopressors, and fluid resuscitation. All patients had a radial artery catheter for pressure monitoring. The side of placement was chosen at the discretion of the treating physician.

Carotid-femoral PWV measurement was performed when patients had achieved mean arterial pressure > 65 mmHg, and there had been no change in vasopressor requirements for at least 1 h. Baseline hemodynamic data (heart rate, systolic, mean, and diastolic blood pressure) were obtained from routine monitoring, and doses of vasopressor agents were recorded. We calculated pulse pressure as the difference between systolic and diastolic arterial pressure.

PWV was measured using a previously tested two-channel photoplethysmograph [19]. Measurement was performed at the patient’s bedside with the ambient light dimmed. With the patient in a supine position, two reflecting photoplethysmograph sensor probes were placed on the skin over the right carotid and femoral arteries and fixed with adhesive tape over an area where maximal arterial pulsation was felt. Once we had obtained a stable arterial waveform from both sensors, the signal was recorded continuously for 2 min. The analog signal from the photoplethysmograph probes was digitized and stored on a computer to be analyzed off-line. The length of the pulse wave path (L) was measured on the surface of the body as the distance between probes located over the carotid and the femoral arteries. To calculate average pulse wave transit time for analysis, we manually chose segments of at least ten consecutive waveforms without movement artefacts. The transit time of the arterial pulse wave (∆T) was measured using the “foot-to-foot” method as the time interval between the upstrokes of two simultaneously recorded arterial waves. PWV in meter per second was calculated using the equation: PWV = L/∆T.

We followed up patients until discharge from hospital or death. Repeated SOFA scores were obtained 48 h after the PWV recording. The results of bacteriological testing were also recorded. The clinical outcomes included in the analyses were hospital mortality, progression of multiple organ failure (defined as increase in the SOFA score of at least 1 point), and improvement of cardiovascular failure (defined as decrease of cardiovascular component of the SOFA score of at least 1 point) over the first 48 h of ICU admission.

### Statistical analysis

We described the demographic, clinical, and hemodynamic characteristics of the patient cohort, stratified by quartiles of PWV distribution. We reported continuous variables as median and inter-quartile range and compared the results by using one-way ANOVA and the Harrell-Davis estimator [20]. Categorical variables were reported as counts and percentages and compared using Fisher’s exact test.

We used a multivariate logistic regression model to assess associations of PWV quartile strata with the progression of multiple organ failure and improvement in the cardiovascular failure in the subgroup of patients who had cardiovascular instability at admission. Vasopressor use was considered as a covariate. The results were reported as adjusted odds ratios and 95% confidence intervals.

We used Cox proportional hazards regression to investigate if patients within different PWV quartiles differed in survival time. Relative hazard ratios were calculated by PWV quartile strata, with and without adjustment for age, APACHE II, and baseline SOFA score.

The model with the best fit was determined by taking into account the likelihood ratio test and by removing covariates with non-significant effects and by checking for change in the PWV quartile estimate. The results were reported as hazard ratios and 95% confidence intervals. A *P* value < 0.05 was considered statistically significant.

All statistical tests were performed in R version 3.3.2 (The R Foundation for Statistical Computing, GNU General Public Licence, MA, Boston, USA) using the survival and WRS2 packages.

## Results

### Study population

**Fig. 1:**
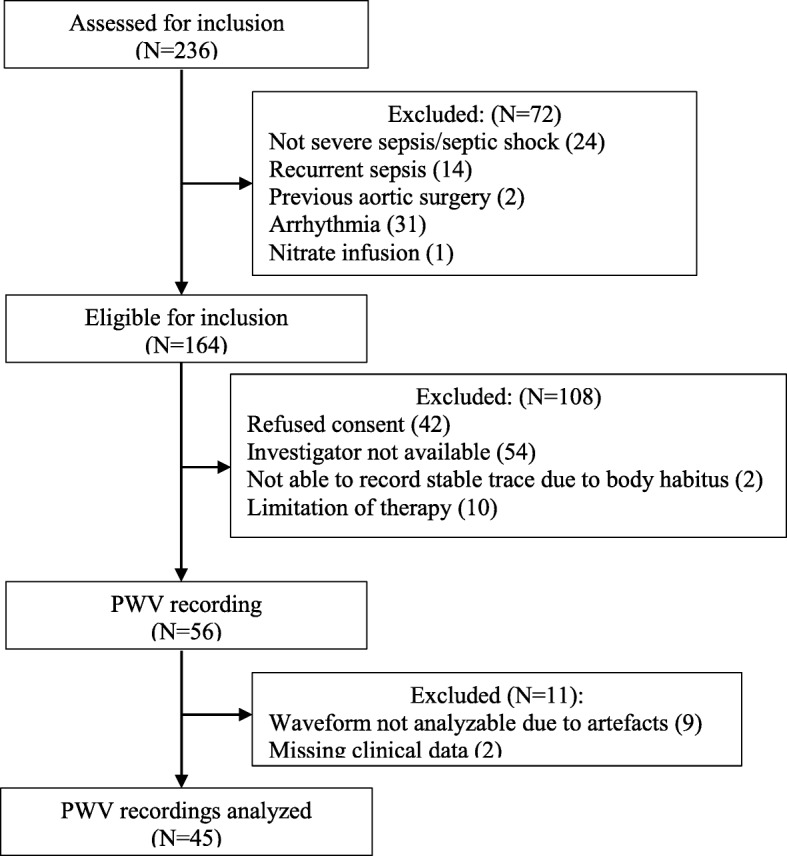
Flow diagram showing inclusion of patients with severe sepsis and septic shock

Of the 164 sepsis patients eligible for inclusion into the study, we obtained simultaneously recorded carotid and femoral pulse waves in 56 individuals. After the exclusion of 11 patients because of movement artefacts, which precluded the measurement of PWV, and missing clinical data, waveforms from 45 patients were available for analysis (Fig. 1). The patients whose recordings were discarded from analyses did not significantly differ from the analyzed patients in terms of age, gender, or disease severity.

The median ICU length of stay was 8 (5–15) days. The source of sepsis was mostly abdominal (36%) or respiratory (33%). Gram-negative bacteria and gram-positive bacteria were involved in 27% and 33% of cases, respectively. At the time of PWV measurement, 31 patients received vasopressors for septic shock. Overall, 11 (24.4%) patients died in the hospital.

The median PWV for the entire sepsis cohort was 14.6 (8.1–24.7) m/s and it exceeded 12 m/s in 25 (55.6%) of the patients in the cohort. We present patient demographic, clinical, and hemodynamic characteristics, stratified by PWV quartile in Table 1.

**Table 1:** Demographic, clinical, and hemodynamic characteristics of the septic patient cohort stratified by their pulse wave velocity quartile

	Total	Pulse wave velocity			
		< 8.1 m/s	8.1–14.6 m/s	14.6–24.7 m/s	> 24.7 m/s
	(*n* = 45)	(*n* = 12)	(*n* = 11)	(*n* = 10)	(*n* = 12)
Demographic characteristics					
Age (in years)	67 (54–75)	61 (43–73)	57 (49–68)	69 (57–79)	59 (39–78)
Gender (females), *n* (%)	17 (38%)	5 (42%)	4 (36%)	5 (50%)	3 (25%)
Clinical characteristics					
APACHE II score	19 (15–25)	21 (14–26)	19 (16–24)	16 (11–19)	19 (15–27)
SOFA score	7 (4–10)	9 (4–10)	8 (5–9)	4 (3–7)	7 (5–11)
CV SOFA	3 (0–3)	1 (0–3)	3(1–3)	0 (0–3)	3 (1–4)
Ventilated, *n* (%)	18 (40%)	6 (50%)	3 (27%)	1 (10%)	8(67%)
Septic shock, *n* (%)	25 (56%)	7 (58%)	6 (55%)	4 (40%)	8 (67%)
Smoker, *n* (%)	18 (40%)	6 (50%)	4 (36%)	3 (30%)	5 (40%)
History of hypertension	21 (47%)	5 (42%)	7 (64%)	6 (56%)	3 (25%)
History of diabetes	5 (11%)	3 (25%)	1 (13%)	1 (11%)	0
Established CAD	3 (7%)	2 (17%)	1 (9%)	0	0
Hemodynamic characteristics					
Systolic blood pressure (mm Hg)	110 (97–121)	113 (107–120)	101 (95–116)	107 (90–128)	108 (97–121)
Mean blood pressure (mmHg)	78 (70–87)	80 (77–85)	75 (70–82)	77 (70–92)	72 (70–82)
Pulse pressure (mmHg)	47 (36–56)	51 (41–53)	48 (34–58)	44 (34–57)	44 (36–56)
Diastolic blood pressure (mmHg)	62 (56–71)	62 (60–71)	66 (55–67)	65 (60–78)	58 (44–66)*
Dose of noradrenaline (mcg/kg/min)	0.03 (0–0.1)	0.03 (0–0.09)	0.03 (0–0.08)	0 (0–0.02)	0.1 (0–0.14)
Heart rate (beats/min)	90 (82–98)	86 (82–94)	91 (87–101)	85 (64–93)	95 (82–98)

Values are given as median (interquartile range) or number (percentage)

*CV SOFA* cardiovascular component of SOFA, *Established CAD* previous myocardial infarction, percutaneous coronary intervention or coronary artery bypass grafting

*Significant at the 0.05 probability level

The second PWV quartile was chosen as the reference group for further analyses as these values fall within the range reported for the general population [21]. Patients in the fourth quartile had lower diastolic pressure than the other groups (*P* = 0.03). Other clinical and hemodynamic characteristics did not differ between PWV quartiles.

### Progression of sepsis by PWV quartile

**Fig. 2:**
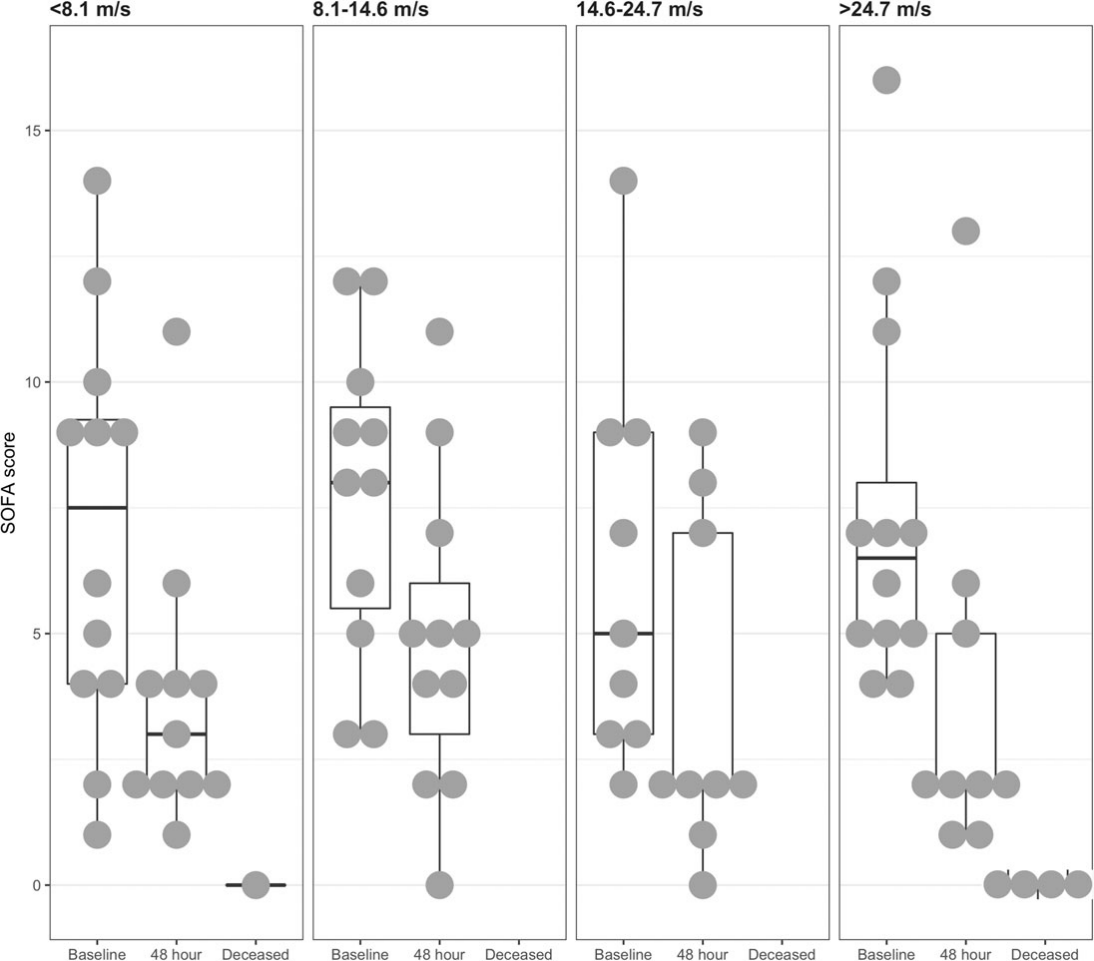
Baseline and 48-h SOFA score comparison stratified by pulse wave velocity quartiles. Five patients died within 48 h, only their baseline SOFA scores could be analyzed

During baseline, assessment patients in all PWV quartiles showed a similar sepsis severity, measured by APACHE II and SOFA scores with a non-significant trend for more severely ill patients being in the first PWV quartile (< 8.1 m/s). Within 48 h of admission to ICU, five patients had died and sequential SOFA scores were available for 40 patients in total. Baseline and 48-h SOFA scores stratified by PWV quartile are shown in Fig. 2.

The association between PWV and the 48-h progression of multiple organ failure (MOF) measured by changes in SOFA scores from the baseline score is shown in Table 2. Compared with the reference PWV quartile (8.1–14.6 m/s), there was no association between PWV and progression of MOF, before or after adjustment for vasopressor use. Although not statistically significant, a larger proportion of patients (33% (first quartile) vs 18, 10, and 25% (second, third, fourth quartiles)) with PWV in the lowest quartile (< 8.1 m/s), had increased SOFA scores over 48 h.

**Table 2:** Association of pulse wave velocity with the evolution of multiple organ and cardiovascular failure

	Unadjusted		Adjusted	
Risk factor	OR (95% CI)	*P* value	OR (95% CI)	*P* value
Progression of MOF				
Carotid-femoral PWV				
< 8.1 m/s	2.57 (0.38–22.76)	0.34	2.50 (0.37–22.71)	0.37
8.1–14.6 m/s	1.0 (reference)	NA	1.00 (reference)	NA
14.6–24.7 m/s	0.64 (0.03–8.13)	0.74	0.62 (0.03–8.3)	0.73
> 24.7 m/s	1.13 (0.11–11.26)	0.92	1.08 (0.11–10.97)	0.94
Use of vasopressors			1.97 (0.40–11.52)	0.42
Improvement of CV failure				
Carotid-femoral PWV				
< 8.1 m/s	2.10 (0.39–12.48)	0.40		
8.1–14.6 m/s	1.0 (reference)	NA		
14.6–24.7 m/s	1.0 (0.16–6.19)	1.0		
> 24.7 m/s	2.63 (0.46–16.78)	0.28		

*MOF* multiple organ failure, *PWV* pulse wave velocity, *CV* cardiovascular, *OR* odds ratio, *CI* confidence interval

### Association between PWV and survival

Overall, 33, 27, and 33% of patients died in hospital in the first, second, and fourth PWV quartile, respectively (*P* = 0.23). All patients in the third PWV quartile survived to hospital discharge.

Cox regression and survival analyses with age, APACHE II, and baseline SOFA as confounders showed a shorter hospital survival time for patients in the highest PWV quartile (> 24.7 m/s) (hazard ratio = 9.45, confidence interval 1.24–72.2; *P* = 0.03), as presented in Additional file 1: Table S1.

## Discussion

When exploring PWV in patients with early sepsis admitted to ICU, we found that high PWV values are prevalent in this population. There was no difference in demographics and disease severity scores across quartiles. Patients in the highest quartile (> 24.7 m/s) had the shortest survival times.

Carotid-femoral PWV is a widely measured vascular biomarker and a strong predictor of future cardiovascular events and mortality in hypertensive patients [22]. It is a composite measure of structural and functional damage in the media of large arteries. An increase in PWV related to structural changes is a result of insults accumulated over time and reflects a loss of elastin and the calcification in the artery wall, usually secondary to subclinical inflammation. In the cardiovascular risk population, non-invasive identification of such structural changes is the main rationale for PWV measurement. In acute inflammation, including sepsis, changes in PWV happen quickly and therefore are most likely due to functional factors, such as vascular smooth muscle tone. Arterial smooth muscle tone depends on sympathetic neural activity via the release of noradrenaline and endothelial function via NO-dependent and independent mechanisms. The PWV in the aorta is also influenced by distending pressure. We expect that this factor has less influence in a cohort of resuscitated sepsis patients. Due to peripheral vasodilatation, their systemic arterial pressures and distending pressure in the aorta are likely to be in the lower range for an individual patient. Indeed, in this cohort of patients, median systolic and diastolic pressures were only 110 and 62 mm Hg, respectively.

In the general population of 60- to 70-year-old normotensive individuals, the 90th percentile of carotid-femoral PWV is 12.2 m/s [21], and values above this are considered a marker of significant alterations of arterial function [23]. In this sample, 55.6% of patients (median age 67 years) had a PWV of more than 12 m/s. Similar increases in PWV have been shown in animal models. In acute endotoxic shock, the central compartment of the vasculature has been shown to become stiffer, but the peripheral appears more compliant, especially in fluid resuscitated animals [24]. Mechanisms for the widespread increase in the PWV in intensive care sepsis populations are not clear, but functional rather than structural causes appear more likely. Sepsis-induced endothelial dysfunction could account for some of the observed changes, but the magnitude of the effect is better explained by a catecholamine excess state caused by sepsis [25]. This is supported by the finding that 67% of patients with a PWV > 24.7 m/s were receiving vasopressors.

We found that an excessively high PWV on admission to the intensive care unit was associated with shorter survival times. If in this group sympathetic overstimulation is, indeed, the main determinant of PWV, detrimental effects of adrenergic stress could explain the finding [26]. In patients with a moderately high PWV, survival times did not differ from that of the reference group. This may be a reflection of a stress response necessary to maintain an adequate perfusion during critical illness with a functional adrenergic response. Further research into the association between PWV and measures of autonomic activity, such as heart rate variability and/or baroreceptor sensitivity in patients with sepsis, could help to understand this phenomenon.

### Limitations of the study

There are several limitations inherent in this study due to the included population and study design. In an investigation involving a single intensive care unit, the number of eligible patients is limited. Therefore, we chose to recruit a heterogenous group of patients with severe sepsis and septic shock. There were major differences between patients in terms of age, use of vasopressors, and the need for sedation and respiratory support. Inclusion of patients with delirium was not possible because of movement artefacts. Baseline cardiovascular risk factors and comorbidities that were important confounders in this context could not be ascertained fully, as the condition of the patients often precluded effective communication.

PWV is a composite measure, which may be affected by rapid fluctuations in blood pressure. Such fluctuations are likely in a cohort of sepsis patients, but we attempted to limit the effects of such fluctuations by including only resuscitated patients who had had no major cardiovascular instability for 1 h.

Considering the small cohort, only robust associations between the progression of MOF and PWV could have been detected. There might be other clinically important associations that we have missed in this study. Patients who died within the 48-h window between the two assessments of SOFA and who had the fastest deterioration of organ function were excluded from the analysis. Consequently, the results are applicable only to patients who survive for at least 2 days after admission to the intensive care unit.

## Conclusions

This study reports a high prevalence of high aortic pulse wave velocity in patients with severe sepsis and septic shock admitted to intensive care. No significant association was found between PWV at ICU admission and the progression of MOF or cardiovascular failure, although analysis shows that the group with PWV > 24.7 m/s had a shorter survival time.

## Additional file

Additional file 1: **Table S1.** Results of Cox regression analysis of the association between pulse wave velocity and length of survival. (DOCX 15 kb)

## Abbreviations

APACHE II: Acute Physiology and Chronic Health Evaluation II
ICU: Intensive care unit
MOF: Multiple organ failure
PWV: Pulse wave velocity
SOFA: Sequential Organ Failure Assessment
