# Supplementary material for: Association between increased arterial stiffness and clinical outcomes in patients with early sepsis: a prospective observational cohort study
Source: Intensive Care Med Exp. 2019 May 16;7:26. doi: 10.1186/s40635-019-0252-3 (PMC6522594; doi:10.1186/s40635-019-0252-3)
Supplement: Supplementary file 1 — Table S1. Results of Cox regression analysis of the association between pulse wave velocity and length of survival. (DOCX 15 kb) [file 40635_2019_252_MOESM1_ESM.docx]

**Additional file 1**

**Table S1** Results of Cox regression analysis of association between pulse wave velocity and length of survival

|  | Unadjusted |  | Adjusted |  |
| --- | --- | --- | --- | --- |
|  | HR (95% CI) | P value | HR (95% CI) | P value |
| *Carotid femoral PWV* |  |  |  |  |
| <8.1 m/s | 1.25 (0.27-5.70) | 0.77 | 1.29 (0.24-6.78) | 0.77 |
| 8.1-14.6 m/s(reference) | 1.0 | NA | 1.0 | NA |
| 14.6-24.7 m/s* | NA | NA | NA | NA |
| >24.7 m/s | 3.89 (0.71-21.00) | 0.11 | 9.45 (1.24-72.2) | 0.03 |
| Age (yrs) |  |  | 1.03 (0.97-1.09) | 0.3 |
| *APACHE II score* |  |  | 0.85 (0.71-1.02) | 0.08 |
| *Admission SOFA score* |  |  | 1.76 (1.14-2.71) | 0.01 |
| PWV: pulse wave velocity, HR: hazard ratio, CI: confidence interval  * No deaths occurred in 3^rd^ PWV quartile | | | | |
